# A case of cola dependency in a woman with recurrent depression

**DOI:** 10.1186/1756-0500-5-692

**Published:** 2012-12-21

**Authors:** Charles Boy Kromann, Connie Thuroee Nielsen

**Affiliations:** 1 Mental Health Services, Region of Southern Denmark, Esbjerg- Ribe, Gl. Vardevej 101, DK-6715, Esbjerg N, Denmark

**Keywords:** Cola caffeinated soft drink, Depression, Addiction, Metabolic syndrome

## Abstract

**Background:**

Cola is an extremely popular caffeinated soft drink. The media have recently cited a poll in which 16% of the respondents considered themselves to be addicted to cola soft drinks. We find the contrast between the apparent prevalence of cola addiction and the lack of scientific literature on the subject remarkable. To our knowledge, this is the first case of cola dependency described in the scientific literature.

**Case presentation:**

The patient is a 40-year-old woman, who when feeling down used cola to give her an energy boost and feel better about herself. During the past seven years her symptoms increased, and she was prescribed antidepressant medication by her family doctor. Due to worsening of symptoms she was hospitalised and later referred to a specialised outpatient clinic for affective disorders. At entry to the clinic she suffered from constant tiredness, lack of energy, failing concentration, problems falling asleep as well as interrupted sleep. She drank about three litres of cola daily, and she had developed a metabolic syndrome.

The patient fulfilled the ICD-10 criteria for dependency, and on the Yale Food Addiction Scale (YFAS) she scored 40 points. Her clinical mental status was at baseline assessed by the Major Depression Inventory (MDI) = 41, Hamilton Depression - 17 item Scale (HAMD-17) = 14, Young Mania Rating Scale (YMRS) = 2 and the Global Assessment of Functioning (GAF) Scale = 45.

During cognitive therapy sessions she was guided to stop drinking cola and was able to moderate her use to an average daily consumption of 200 ml of cola. Her concentration improved and she felt mentally and physically better. At discharge one year after entry her YFAS was zero. She was mentally stable (MDI =1, HAMD-17 = 0, YMRS = 0 and GAF = 85) and without antidepressant medication. She had lost 7.2 kg, her waistline was reduced by 13 cm and the metabolic syndrome disappeared.

**Conclusion:**

This case serves as an example of how the overconsumption of a caffeinated soft drink likely was causing or accentuating the patient’s symptoms of mental disorder. When diagnosing and treating depression, health professionals should pay attention to potential overuse of cola or other caffeinated beverages.

## Background

The mental disorder recurrent depression or depressive episode is in ICD-10 [1] characterised by the following core symptoms: depressed mood, lack of energy and decreased interest. Accompanying symptoms include decreased appetite, problems with concentration and sleep disturbance [1]. Untreated, the condition will often result in improper diurnal rhythm, disturbances in eating habits and for some increased use of tobacco [2], alcohol or other stimulants [3], e.g. caffeine. This self-medicating behaviour can be seen as both comfort-seeking and as a compensation for lack of energy [4].

Cola is an extremely popular caffeinated soft drink. Some brands have secret recipes. The most popular brand has a declared sugar content of 106 g/L [5] and a caffeine concentration of about 100 mg/L [6]. Lately, the media have presented articles about cola-addicts, and recently one Danish radio station cited a poll in which 16% of 1006 respondents considered themselves to be addicted to cola [7]. A search on the internet revealed that there was a surprisingly large number of private practice rehab therapists offering their services to cola addicts [8].

Psychiatric patients often have addictive behaviours, and a study by O’Farrell states that 90% of all hospitalised psychiatric patients show signs of addictive behaviour [9]. The scientific literature on addictive behaviour related to intake of cola is to our knowledge non-existing. Various searches in the scientific literature as well as more general open literature using PubMed, Google Scholar on “cola” and “dependency” or “addiction” yield no papers on addiction to cola, although some papers on addiction to caffeine mention cola as a caffeine source. Caffeine itself is a well-described substance of abuse and ICD-10 contains definitions for both dependence and withdrawal symptoms [1,10].

It is good clinical practice to consider abuse of alcohol and drugs when treating psychiatric patients, but patients are rarely asked about consumption of soft drinks containing caffeine.

We find the contrast between the belief that cola addiction exists and the apparent lack of scientific literature on the subject interesting and present the case of a 40-year-old woman with recurrent depression, who considered herself addicted to cola and whose daily consumption of cola increased during depressive episodes. To the best of our knowledge, this is the first case of cola dependency described in the scientific literature.

## Case presentation

The patient is a 40-year-old woman, who never drank coffee, rarely drank alcohol, but smoked 20 cigarettes per day. She worked as a waitress from the age of 21 years and had unlimited access to cola. During long work shifts she frequently used cola to boost her energy. When she later became a mother, she, as a role model, tried to cut down on cola consumption. However, when she felt down she often used cola to give her an energy boost and thus feel better about herself. Apart from her self-reported addiction to cola and cigarettes she had never used or been addicted to other substances.

### Symptoms

The patient had been taking antidepressant medication for seven years. Her family doctor had treated her the first two years with citalopram and with duloxetine, 120 mg, for the last five years. Due to worsening of symptoms (suicidal intentions, lack of energy and sleep disturbances) she was hospitalised for four weeks. She was subsequently discharged with the antidepressant duloxetine, 120 mg, and additional medication (quetiapine, 75 mg and zopiclone, 10 mg) and referred to a specialised outpatient clinic for affective disorders. On admission she still suffered from constant tiredness, lack of energy and failing concentration, and could hardly get her children to school. She had also had a constant feeling of restlessness and difficulties falling asleep, as well as interrupted sleep. Asked about eating habits, she revealed that she drank about three litres of a specific cola brand daily. She had over the years tried other cola brands, but these brands could not give her the same kick of energy feeling. She explained that after a cola intake her tiredness would shortly disappear. Her craving for cola was so pronounced that she fulfilled the ICD-10 criteria for dependency [1], and on the Danish translation of the Yale Food Addiction-scale (YFAS) [11] she scored 40 points.

Her clinical mental status was at baseline assessed by the Major Depression Inventory (MDI) =41, Hamilton Depression - 17 item Scale (HAMD-17) =14, Young Mania Rating Scale (YRMS) =2, Global assessment of functioning (GAF) =45 and the physical status by waist circumference =101 cm, weight= 72.9 kg, blood pressure=108/75 mmHg. Laboratory test: Fasting blood glucose: 5.9 mmol/l, HDL: 1.17 mmol/l, triglycerides: 0.75 mmol/l. She fulfilled the criteria for metabolic syndrome according to the International Federation of Diabetes (IDF) [12].

She was offered cognitive therapy according to the guidelines for recurrent depression. The lack of energy and the feeling of guilt in relation to her parenthood were some of the themes in her case formulation. During sessions she was informed that her excessive consumption of cola could “negatively affect her brain”. She decided to stop drinking cola completely. However, shortly after, she was obsessing over cola and had craving for the soft drink. She was then guided to reduce the consumption and dilute the cola with ice cubes. The following six months she was able to reduce her use to an average daily consumption of 200 ml of cola. During these six months her concentration skills improved, and she felt mentally as well as physically better, so in collaboration with her psychiatrist at the clinic she decided to reduce her psychopharmacological medication.

### Treatment outcome

At discharge from the outpatient clinic a year after entry she still had an average daily intake of 200 ml cola but her YFAS-score was now zero. She was not taking any medication and she was mentally stable and assessed by MDI=1, HAM-17=0, YMRS=0, GCI=2 and GAF=85. Due to the reduced consumption of soft drinks she lost weight to 65.7 kg, her waist circumference was reduced to 88 cm, and her blood pressure was approximately the same, 109/77 mmHg. Laboratory test: Fasting blood glucose was reduced to 4.3 mmol/l, HDL: 1.13 mmol/l, triglycerides: 0.78 mmol/l, and she no longer fulfilled the criteria for metabolic syndrome according to IDF.

## Discussion

We have presented a case of cola dependency in a woman with recurrent depression. As part of the treatment for her mental disorder her cola dependency was addressed and treated, with good effect on both her mental and physical health. The fact that her dependence on cola existed ahead of her recurrent depression, and that she could not reduce her consumption of cola by herself, is not sufficient to conclude that her dependence on cola was the primary cause of her recurrent depressions. It is, however, remarkable that both her dependence on cola and her depressive symptoms were reduced simultaneously, when she was treated with a recommended therapeutic method.

Caffeine is the world’s most widely consumed psychoactive substance and has associated psychiatric syndromes such as caffeine-induced sleep disorder, caffeine-induced anxiety disorder and caffeine dependency with withdrawal symptoms that are all well described and documented in the literature [13].

A daily caffeine intake as low as 100 mg caffeine (1 cup of coffee or one litre of cola may result in caffeine dependency and subsequently withdrawal symptoms such as headaches, drowsiness, dysphoric mood, depression and concentration difficulties [13].

It is straightforward to consider the patients symptoms solely to be related to caffeine dependency, but several aspects of the case point to a concept of cola dependency separate from the previously described caffeine dependency.

The patient had a distinct preference for a specific band of cola. Although she repeatedly tried, she could not find a satisfactory replacement for her preferred drink, not even among other caffeinated cola-flavoured soft drinks. Caffeine dependency is shown to be associated with taste preferences in the withdrawal phase, but only in acute caffeine abstinence [14]. The patient slowly reduced her consumption to 200 ml of cola equivalent to 25 mg of caffeine, but was unable to substitute the cola or stop the use completely.

It is very plausible that there is a synergistic effect of caffeine and sugar in drinks, as most energy drinks boast this combination. This effect could add to the addictiveness of cola as sugar is also attributed an addictive potential [15].

The most interesting aspect of the argumentation for the concept of a separate cola addiction is the common belief in cola dependency among lay people. More than every seventh person in Denmark consider themselves addicted to cola [7], some to such an extent that they are willing to pay for treatment in order to reduce their consumption.

Whilst the aforementioned poll with self-evaluated dependency is statistically sound, it is probably not scientifically valid. However, the large number of people who consider themselves addicted is still a relevant indication that a potential problem exists.

Cola and other caffeinated soft drinks are not associated with immediate health-threatening effects. They may, however, be related to prolonged and extensive consumption, mainly due to the high sugar content and thus the risk of obesity-related diseases. With regard to dependence on cola there is a discrepancy between the public perception and the official perception among health professionals. This may result in patients having problems with addiction to cola not getting attention in relation to their symptoms, or proper preventive or therapeutic treatment in relation to reduction of their consumption. Individual counseling on this matter could therefore be lacking. This lost opportunity for individual counseling could have negative consequences for patients, who are not reachable through health campaigns.

A case report is no proof of the existence of a specific cola addiction, and more research must be carried out in this field, in order to shed light on the apparent discrepancy between popular belief and the official more reluctant perception among health professionals. Studies examining the potential soft drink addiction are needed.

## Conclusion

This case also serves as an example, where the overconsumption of a caffeinated soft drink most probably caused or accentuated the patient’s symptoms of mental disorder. When diagnosing and treating depression, health professionals should pay attention to potential overuse of cola or other caffeinated beverages.

## Consent

Written and informed consent from the patient was obtained for the publication of this case report.

## Competing interests

The authors declare that they have no competing interests.

## Authors’ contributions

CBK conducted the literature review and was a major contributor in the writing of the manuscript. CTN supervised data collection and was a major contributor in the writing of the manuscript. All authors read and approved the final manuscript.
